# A novel epitope-presenting thermostable scaffold for the development of highly specific insulin-like growth factor-1/2 antibodies

**DOI:** 10.1074/jbc.RA119.007654

**Published:** 2019-07-23

**Authors:** Carmen Peeß, Christian Scholz, David Casagolda, Hartmut Düfel, Michael Gerg, Frank Kowalewsky, Marco Bocola, Leopold von Proff, Sabine Goller, Heidi Klöppel-Swarlik, Alessandra Hoppe, Michael Schräml

**Affiliations:** ‡ Antibody Development, Roche Diagnostics GmbH, 82377 Penzberg, Germany; § Protein Design, Roche Diagnostics GmbH, 82377 Penzberg, Germany; ¶ Enzyme & Protein Technologies, Roche Diagnostics GmbH, 82377 Penzberg, Germany; ‖ Lehrstuhl für Biotechnologie, RWTH Aachen University, 52074 Aachen, Germany; ** Endocrinological Diseases III, Centralized and Point of Care, Roche Diagnostics GmbH, 82377 Penzberg, Germany

**Keywords:** antibody, antigen presentation, chaperone, insulin-like growth factor (IGF), protein engineering, antigen mimic, FK-506 binding protein, FKBP domain, immunogen

## Abstract

High sequence and structural homology between mature human insulin-like growth factors IGF-1 and IGF-2 makes serological discrimination by immunodiagnostic IGF tests a challenging task. There is an urgent need for highly specific IGF-1 and IGF-2 antibodies, yet only a short sequence element, *i.e.* the IGF loop, provides enough difference in sequence to discriminate between the two molecules. We sought to address this unmet demand by investigating novel chimeric immunogens as carriers for recombinant peptide motif grafting. We found *Thermus thermophilus* sensitive to lysis D (SlyD) and *Thermococcus gammatolerans* SlyD FK-506–binding protein (FKBP) domains suitable for presentation of the predefined epitopes, namely the IGF-1 and IGF-2 loops. Chimeric SlyD-IGF proteins allowed for the development of exceptionally specific IGF-1 and IGF-2 monoclonal antibodies. The selected antibodies bound with high affinity to the distinct IGF epitopes displayed on the protein scaffolds, as well as on the mature human IGF isoforms. The respective SlyD scaffolds display favorable engineering properties in that they are small, monomeric, and cysteine-free and can be produced in high yields in a prokaryotic host, such as *Escherichia coli*. In conclusion, FKBP domains from thermostable SlyD proteins are highly suitable as a generic scaffold platform for epitope grafting.

## Introduction

Insulin-like growth factors IGF-1^2^ and IGF-2 are part of the IGF axis, a complex endocrine system that regulates cell growth and development. Mature human IGF-1 is a 70-amino acid polypeptide hormone (7.7 kDa) that is critical for normal body growth and development (1, 2). Deficiency or elevation of IGF-1 levels are associated with various diseases including growth failure or acromegaly and gynecological cancers, respectively (3). Mature IGF-2 is a 67-amino acid peptide (7.5 kDa), which shares large stretches of sequence identity (55%) and a high degree of structural homology with IGF-1 (4, 5). Homology between these molecules and the presence of IGF-2 at a 3-fold molar excess over IGF-1 in human serum (6, 7) imply that the specific detection and quantitation of IGF-1 by immunodiagnostic tests are difficult. As a consequence, highly specific antibodies that are devoid of immunological IGF-2 cross-reactivity and can cope with the presence of the at least equally concentrated potential interfering factor IGF-2 in serum are a prerequisite for IGF-1–specific diagnostic assays. Conversely, the same holds true for IGF-2 serology in the presence of IGF-1.

In principle, immunological discrimination between IGF-1 and IGF-2 should be feasible when the respective antibody specifically targets an IGF-1 epitope that differs in either amino acid sequence or conformation from its IGF-2 counterpart. Indeed, there is only one obvious difference in sequence between IGF-1 and IGF-2, confined to the turn–loop motifs at amino acid positions 74–90 (IGF-1_human, UniProtKB entry P05019) and 53–65 (IGF-2_human, UniProtKB entry P01344), respectively. To the best of our knowledge, it has hitherto not been possible to develop antibodies targeting this loop motif by conventional immunization strategies using native IGFs or IGF-derived peptides. Because both IGF isoforms share very high sequence homology between species, including mice, it is also challenging to overcome the self-tolerance barrier (8). Immunization of animals with keyhole limpet hemocyanin–grafted IGF peptide has previously been explored and generated antibodies with incomplete IGF epitope coverage. The respective loop sequences were not targeted, possibly because of a lack of immunogenicity or accessibility (9, 10); however, differential phage display panning resulted in the successful generation of anti–IGF-2–specific human antibodies, showing no cross-reactivity toward IGF-1 (11).

Recombinant insertion of desired epitopes into the permissive site of a heterologous protein scaffold is a novel approach to overcome the issues described above. We investigated a scaffold platform based on the sensitive to lysis *D* (SlyD) protein, a product of the *SlyD* gene. SlyD is a member of the FKBP family of prolyl isomerases (12, 13). It is a two-domain protein comprising a larger FKBP domain harboring peptidyl-prolyl *cis*/*trans* isomerase activity, and a smaller insert-in-flap (IF) domain with chaperone functions (14, 15). The poor folding activity of the catalytic FKBP domain is strongly enhanced when it is combined with a polypeptide-binding chaperone domain (16, 17).

The principle of balanced interplay between chaperone and enzyme domains is realized in many natural folding enzymes such as trigger factor (18), FkpA (19, 20), and SlyD (13). Most intriguingly, it turned out to be feasible to create efficient folding enzymes according to this seemingly simple combinatorial blueprint: transfer of the IF domain from *Escherichia coli* SlyD into the flap region of human FKBP12 yielded an artificial folding enzyme with outstanding catalytic properties in protein folding (16, 17). Moreover, it was even possible to graft unrelated chaperone domains such as the apical domain from GroEL or the chaperone domains from yeast protein disulfide isomerase or prokaryotic SurA onto human FKBP12 and thus to design artificial folding enzymes with outstanding properties when compared with the parent FKBP12 molecule (21). Based on these findings, we reasoned that it should be worthwhile to not only use the FKBP domain as a platform to engineer improved artificial folding helpers through combinatorial approaches (16, 21, 22) but also to use the FKBP domain as a scaffold for the display of immunogens. However, human FKBP12 turned out to be only marginally stable and thus not able to provide enough stability to accommodate an insert sequence. As an alternative, FKBP domains from extremophilic organisms are supposed to possess a much higher stability, so we investigated the FKBP domains of *Thermus thermophilus* SlyD (*Tt*SlyD-wt) and *Thermococcus gammatolerans* SlyD (*Tg*SlyD-wt) for use as immunogen scaffolds (23–25).

## Results

### Scaffold design and molecular dynamic (MD) simulations

**Figure 1.**
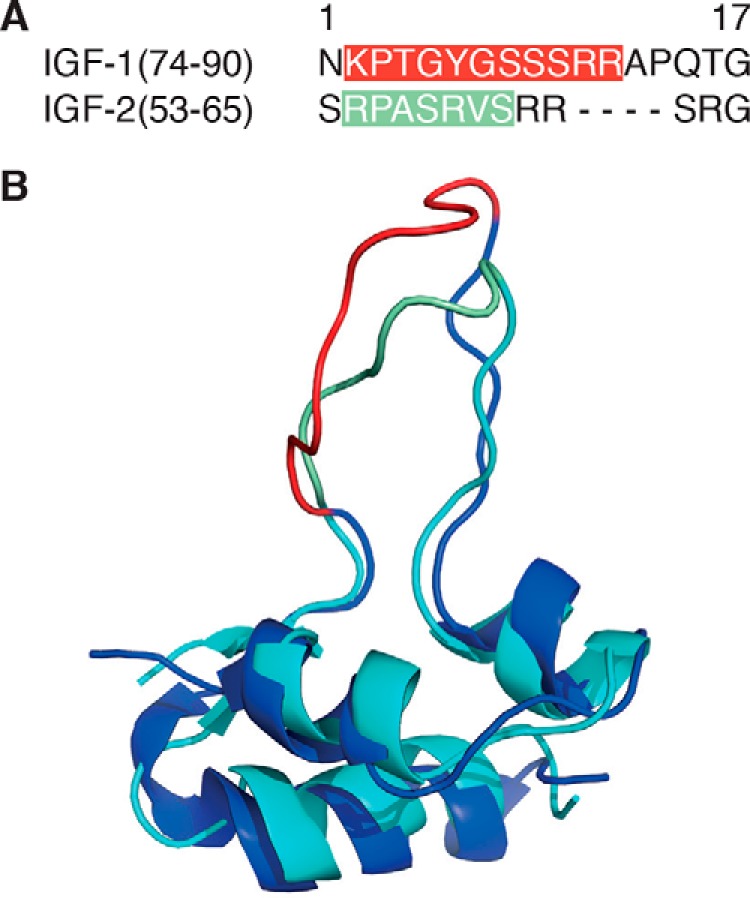
**Sequence alignment IGF-1(74–90) and IGF-2(53–65).** Shown is the structure comparison of IGF-1 and IGF-2. *A*, antibody epitopes that contributed to the immunologic discrimination between IGF-1 and IGF-2 are highlighted in *red* and *green* in the sequence alignment. *B*, the respective antibody epitopes in the loop segments are marked *red* in IGF-1 and *green* in IGF-2. The superimposition of IGF-1 (*dark blue*, RCSB PDB entry 1PMX) and IGF-2 (*light blue*, RCSB PDB entry 1IGL) was arranged with the PyMOL molecular graphics software 1.6 (Schrödinger).

**Figure 2.**
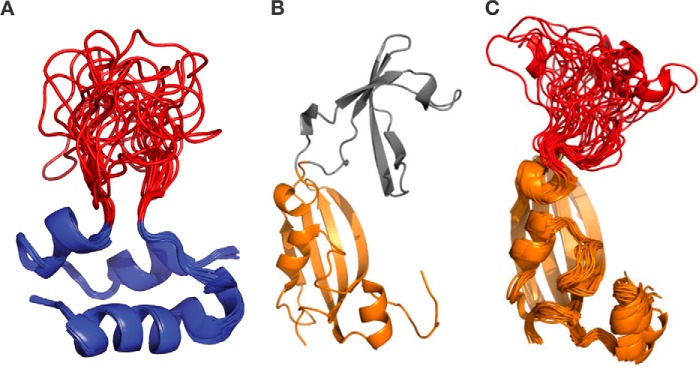
**Concept of the SlyD scaffold technology.** Comparison of IGF-1 loop (*red*) mobility in IGF-1 solution NMR structure bundle (RCSB PDB entry 1PMX; *A*), *Tt*SlyD FKBP scaffold (*orange*; RCSB PDB entry 3CGM) with IF domain (*gray*, *B*); and 40-ns MD simulations structure bundle of the IGF-1 loop (*red*) grafted onto the *Tt*SlyD FKBP scaffold protein (*C*). The figure was drawn in cartoon representation using Visual Molecular Dynamics (VMD, http://www.ks.uiuc.edu/Research/vmd/; please note that the JBC is not responsible for the long-term archiving and maintenance of this site or any other third party hosted site) (27, 39) and PyMOL molecular graphics software 1.6 (Schrödinger).

Structural comparison revealed a notable sequence and structure difference between IGF-1 and IGF-2, namely a distinct loop structure with high flexibility in amino acid position 74–90 (IGF-1) and 53–65 (IGF-2; Fig. 1), respectively. A molecular model was constructed by replacing the IF-domain structure of *T. thermophilus* SlyD (23) by the 1.9-kDa loop sequence ^74^NKPTGYYGSSSRRAPQTG^90^ from IGF-1(74–90). The MD simulations indicated that a linker type with three amino acid residues flanking the insertion site might generate too much graft flexibility and would allow the IGF-1 loop to transiently interact with residues from the FKBP fold (data not shown). Using a single glycine residue connecting the IGF-1(74–90) loop (Table 1) to the FKBP domain avoided this unwanted potential side effect. The 13-amino acid residue IGF-2(53–65) loop sequence, ^53^SRPASRVSRRSRG^65^, was integrated into the *Tt*SlyD FKBP domain accordingly (Fig. 2). Because no structural information on *Tg*SlyD was available, the insertion site design was guided by mere sequence homology considerations. Furthermore, reference constructs were generated by replacing the IF domains by the shortcut linker sequence GAGSGSSG to yield *Tt*SlyD-ΔIF and *Tg*SlyD-ΔIF.

**Table 1:** **SlyD FKBP scaffold amino acid sequences** Ct-SlyD-FKBP, sequence downstream of insertion site; L, linker; Nt-SlyD-FKBP, sequence upstream of insertion site; *Tg*SlyD, *T. gammatolerans* SlyD; *Tt*SlyD, *T. thermophilus* SlyD.

SlyD type	kDa	Nt-SlyD-FKBP	L	Insert	L	Ct-SlyD-FKBP
TtSlyD-wt	17.7	MKVGQDKVVTIRYTLQVEGEVLDQGELSYLHGHRNLIPGLEEALEGREEGEAFQAHVPAEKAYGPH		DPEGVQVVPLSAFPEDAEVVPGAQFYAQDMEGNPMPLTVVAVEGEEVTVDFNHPL		AGKDLDFQVEVVKVREATPEELLHGHAHGGGSRKHHHHHH
*Tt*SlyD-IGF-1(74–90)	14.2	MRGSKVGQDKVVTIRYTLQVEGEVLDQGELSYLHGHRNLIPGLEEALEGREEGEAFQAHVPAEKAYGPH	G	NKPTGYGSSSRRAPQTG	G	AGKDLDFQVEVVKVREATPEELLHGHAHGGGSRKHHHHHHHH
*Tt*SlyD-IGF-2(53–65)	14	MRGSKVGQDKVVTIRYTLQVEGEVLDQGELSYLHGHRNLIPGLEEALEGREEGEAFQAHVPAEKAYGPH	G	SRPASRVSRR SRG	G	AGKDLDFQVEVVKVREATPEELLHGHAHGGGSRKHHHHHHHH
TtSlyD-ΔIF	12.9	MRGSKVGQDKVVTIRYTLQVEGEVLDQGELSYLHGHRNLIPGLEEALEGREEGEAFQAHVPAEKAYGPH	G	AGSGSS	G	AGKDLDFQVEVVKVREATPEELLHGHAHGGGSRKHHHHHHHH
*Tg*SlyD-wt	18.6	MKVERGDFVLFNYVGRYENGEVFDTSYESVAREQGIFVEEREYSPIGVTVGAGEIIPGIEEALLGMELGEKKEVVVPPEKGYGMP		REDLIVPVPIEQFTSAGLEPVEGMYVMTDAGIAKILKVEEKTVRLDFNHPL		AGKTAIFEIEVVEIKKAGEAGGGSRKHHHHHHHH
*Tg*SlyD-IGF-2(53–65)	14.7	MKVERGDFVLFNYVGRYENGEVFDTSYESVAREQGIFVEEREYSPIGVTVGAGEIIPGIEEALLGMELGEKKEVVVPPEKGYGMP	G	SRPASRVSRRSRG	G	AGKTAIFEIEVVEIKKAGEAGGGSRKHHHHHHHH
*Tg*SlyD-ΔIF	13.7	MKVERGDFVLFNYVGRYENGEVFDTSYESVAREQGIFVEEREYSPIGVTVGAGEIIPGIEEALLGMELGEKKEVVVPPEKGYGMP	G	AGSGSS	G	AGKTAIFEIEVVEIKKAGEAGGGSRKHHHHHHHH

### Scaffold preparation: expression and purification

Expression of SlyD chimeric fusion proteins in *E. coli* resulted in high production yields (Table S1). Simple matrix-assisted refolding and purification takes advantage of the robust refolding capabilities of the SlyD scaffold domains. Final polishing of the SlyD variants by size-exclusion chromatography separated the monomeric SlyD fraction from small amounts of apparent dimers and higher associates (Fig. S1). Analysis of the purified monomeric SlyD proteins by SDS-PAGE and analytical size-exclusion chromatography showed single discrete bands migrating at the appropriate molecular weight (Fig. S2). The monomeric scaffold proteins were stable and soluble at 4 °C and at ambient temperature (data not shown).

### CD spectroscopic measurements

**Figure 3.**
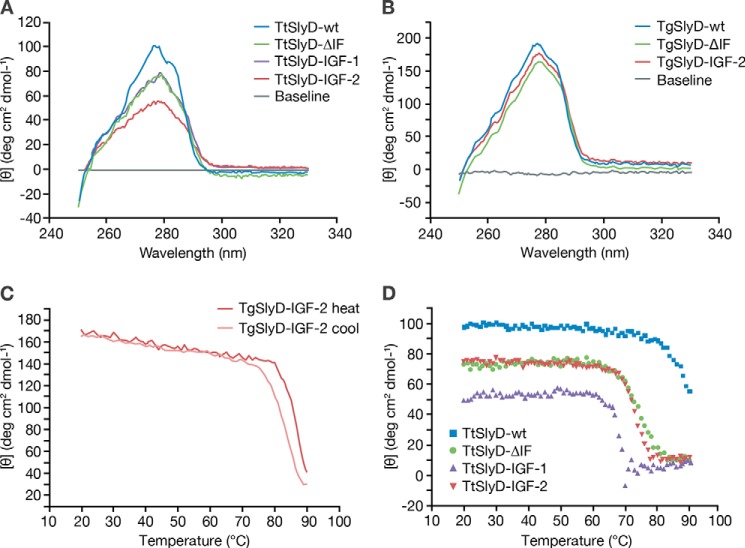
**Near UV CD of TtSlyD and TgSlyD derivatives.** *A*, near-UV CD scans of the *T. thermophilus* SlyD derivatives *Tt*SlyD-wt, *Tt*SlyD-ΔIF, *Tt*SlyD-IGF-1(74–90), and *Tt*SlyD-IGF-2(53–65) at 20 °C. *B*, near UV scans of *T. gammatolerans* SlyD derivatives *Tg*SlyD-wt, *Tg*SlyD-ΔIF, and *Tg*SlyD-IGF-2(53–65) at 20 °C. *C*, reversible temperature transition of *Tg*SlyD-IGF-2(53–65), monitored during instrument heating and subsequent cooling cycle. *D*, temperature transitions of the *Tt*SlyD derivatives *Tt*SlyD-wt, *Tt*SlyD-ΔIF, *Tt*SlyD-IGF-1(74–90), and *Tt*SlyD-IGF-2(53–65) in a temperature gradient from 20 to 95 °C.

To investigate the structural properties and stability of several scaffold monomers, we compared the near UV signatures obtained by CD spectroscopy of *Tt*SlyD-wt and *Tt*SlyD-ΔIF with *Tt*SlyD-IGF-1(74–90) and *Tt*SlyD-IGF-2(53–65) and of *Tg*SlyD-wt and *Tg*SlyD-ΔIF with *Tg*SlyD-IGF-2(53–65), respectively. All SlyD variants exhibited near-UV CD spectra indicative of a native-like folded, compact structure of the FKBP domain (Fig. 3).

*Tt*SlyD-wt remained in a stable folded conformation up to 80 °C, and partially unfolded until 90 °C. The baseline of the denatured protein could not be reached in the accessible temperature range of the unfolding experiment (Fig. 3). *Tt*SlyD-ΔIF was stable up to 62 °C. It showed a midpoint of the unfolding transition at ∼70 °C and was completely unfolded at ∼85 °C. *Tt*SlyD-IGF-1(74–90) was stable up to 60 °C, unfolds at temperatures between 61–65 °C, and seemed to aggregate at higher temperatures. *Tg*SlyD-wt maintained its native baseline up to 76 °C, whereas *Tg*SlyD-ΔIF retained its native conformation up to 80 °C (data not shown). The graft-bearing variant *Tg*SlyD-IGF-2(53–65) was stable up to 78 °C (Fig. 3). It is noteworthy that, after thermally induced partial unfolding up to 90 °C, the *Tg*SlyD-IGF-2(53–65) graft variant was able to fold back reversibly when the protein solution was chilled from 90 to 20 °C. The high thermal stability of the WT scaffold proteins was generally somewhat lowered by the grafted insert but was sufficient for the robust production of stable immunogens and screening reagents.

### Generation of monoclonal antibodies (mAbs)

To develop site-specific antibodies against the IGF-1 and IGF-2 loop segments, respectively, the thermostable scaffold proteins *Tt*SlyD-IGF-1(74–90) and *Tg*SlyD-IGF-2(53–65) were used as immunogens in mice. Table S2 shows the titers in Naval Medical Research Institute mice. The *Tt*SlyD-IGF-1 immunized mice developed measurable IGF-1 loop-specific titers. The *Tg*SlyD-IGF-2 immunized mice developed titers *versus Tg*SlyD-IGF-2 and *Tg*SlyD-ΔIF. Using *Tt*SlyD-IGF-2 and *Tt*SlyD-wt as counter screening reagents IGF-2 loop-specific titers could be confirmed. The relative titers indicate that thermostable SlyD-IGF scaffold proteins can be used as immunogen surrogates and as screening reagents for epitope targeting purposes. The anti–IGF-1 antibody heavy- and light-chain variable domain sequences are listed in Fig. S3.

### Kinetic characterization: surface plasmon resonance analyses

**Figure 4.**
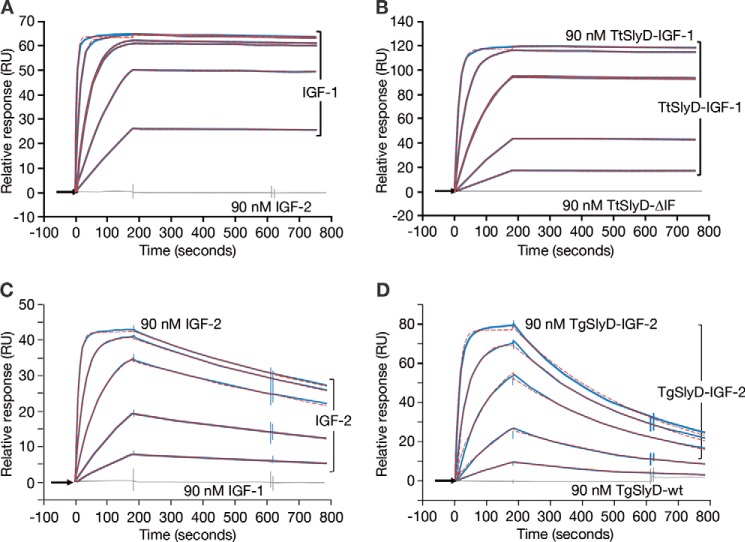
**Biacore sensorgrams showing mAb anti–IGF-1 and mAb anti-IGF2 kinetic profiles.** Kinetic data are in *blue*, overlaid by a 1:1 Langmuir fitting model in *red*, and control injections are in *gray. A*, IGF-1 concentration series *versus* mAb anti–IGF-1 and IGF-2 control. *B*, *Tt*SlyD-IGF-1 concentration series *versus* mAb anti-IGF1 and *Tt*SlyD-ΔIF control. *C*, IGF-2 concentration series *versus* mAb anti–IGF-2 and IGF-1 control. *D*, *Tg*SlyD-IGF-2 concentration series *versus* mAb anti–IGF-2 and *Tg*SlyD-wt control.

**Figure 5.**
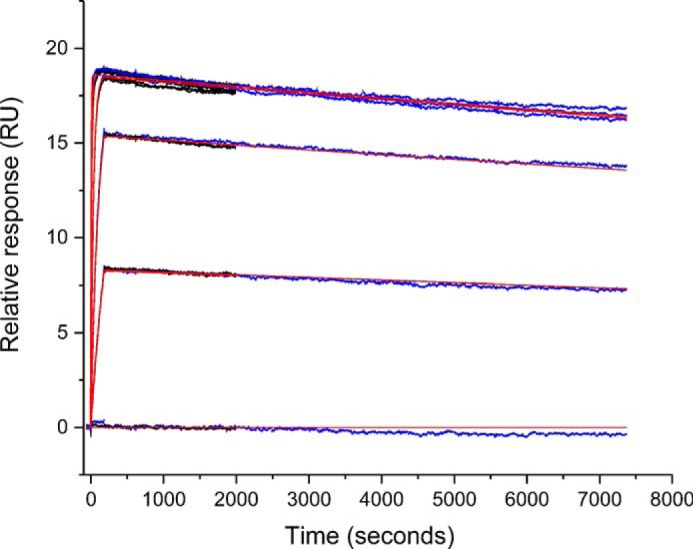
**Biacore mAb anti IGF-1(74–90) kinetics.** *Blue*, 120-min dissociation time; *black*, 30-min dissociation time; *red*, overlaid 1:1 Langmuir fitting model. 90 nm IGF-1 concentration was analyzed in triplicate. For kinetic parameters, see Table 2.

We tested several commercially available anti-IGF mAbs, but all were disqualified because of considerable fast-on/-off kinetics *versus* the respective counterpart IGF isoform (data not shown). Kinetic data of the finally selected mAb IGF-1(74–90) and mAb IGF-2(53–65) are listed in Table 2. Sensorgrams, exemplifying affinity and specificity measurements, are depicted in Fig. 4. The mAb anti–IGF-1(74–90) was quantified by a 120-min dissociation time experiment (Fig. 5) with *K_D_* 6 pm IGF-1 affinity with *k_a_* 3.0E+06 m^−1^ s^−1^ association rate constant and dissociation rate constant *k_d_* 1.7E-05 s^−1^ and *t*_½ diss_ 680 min. The mAb anti–IGF-2(53–65) was quantified with *K_D_* 0.7 nm IGF-2 affinity with *k_a_* 1.2E+06 m^−1^ s^−1^ and *k_d_* 7.7E-04 s^−1^ and *t*_½ diss_ 15 min. IGF control injections clearly show no signal response of mAb anti–IGF-1(74–90) with IGF-2, and, conversely, of mAb anti–IGF-2(53–65) with IGF-1. IGF loop specificities were assessed by using scaffold analytes as indicated in Table 2. Both mAbs show subnanomolar affinity *K_D_* (m) toward their prime targets IGF-1 and IGF-2. Both mAbs showed graft-specific high affinity toward their scaffold immunogens; the empty control scaffolds *Tt*SlyD-ΔIF and *Tg*SlyD-ΔIF were neither recognized nor bound at all.

**Table 2:** **Kinetic characterization of anti–IGF-1 and anti–IGF-2 monoclonal antibodies** ND, not determinable; *Tg*SlyD, *T. gammatolerans* SlyD; *Tt*SlyD, *T. thermophilus* SlyD.

Ligand mAb	Analyte (*n* = 3)	*K_a_*	S.E. (*k_a_*)	*k_d_*	S.E. (*k_d_*)	*K_D_*	S.E. (*K_D_*)
		*m*^−*1*^ *s*^−*1*^		*s*^−*1*^		*m*	
Anti–IGF-1(74–90)	IGF-1*^a^*	3.0E + 06	1.8E + 02	1.7E-05	1.1E-09	5.5E-12	6.1E-14
	IGF-2	ND	ND	ND	ND	ND	ND
	*Tt*SlyD-IGF-1	9.2E + 05	6.6E + 02	2.8E-05	8.6E-08	3.1E-11	1.31E-12
	*Tt*SlyD-ΔIF	ND	ND	ND	ND	ND	ND
Anti–IGF-2(53–65)	IGF-1	ND	ND	ND	ND	ND	ND
	IGF-2	1.2E + 06	1.1E + 03	7.7E-04	3.0E-07	6.6E-10	6.8E-13
	*Tg*SlyD-IGF-2 (53–65)	8.7E + 05	1.1E + 03	2.2E-03	1.5E-06	2.5E-09	3.6E-12
	*Tg*SlyD-wt	ND	ND	ND	ND	ND	ND

*^a^* Determined by 120-min dissociation kinetics (see Fig. 5).

### Peptide array epitope mapping

Peptide-based 2D epitope mapping revealed that both antibody recognition sites were confined to the designated IGF loop sequences (Fig. S4). The anti–IGF-1 antibody binds to an IGF-1 linear sequence stretch comprising the amino acid residues Lys^75^ to Arg^84^. Based on an alanine scan, Gly^78^, Tyr^79^, Gly^80^, Ser^83^, and Arg^84^ were identified as key epitope residues: ^75^KPT*GYG*SS*SR*^84^. The anti–IGF-2 antibody binds to the amino acid sequence Arg^54^ to Ser^60^ within the IGF-2 loop motif. The alanine scan revealed Ser^57^ and Val^59^ as essential residues: ^54^*R*PASRV*S*^60^.

### ECL interference study

**Figure 6.**
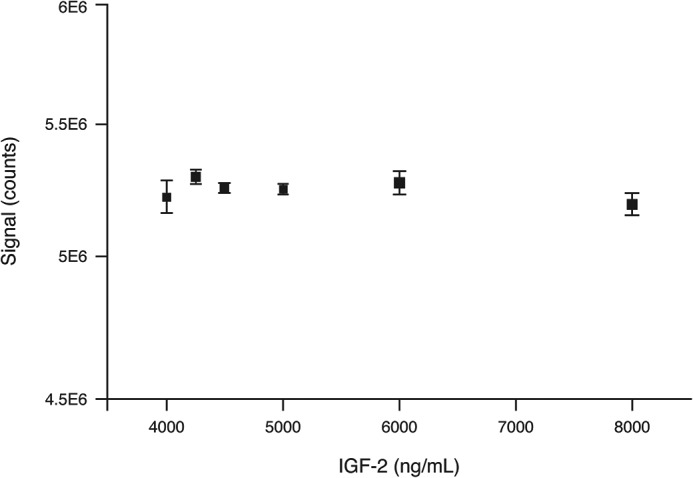
**IGF-1 ECLIA measurement in presence of excess IGF-2.** The samples were supplemented with a constant 1 μg/ml IGF-1 concentration and spiked with increasing IGF-2 concentrations.

Based on electrochemiluminescence immunoassay (ECLIA) measurements, we showed that addition of recombinant-derived IGF-2 does not interfere with the IGF-1 signal response between 4 and 8 μg/ml IGF-2 when using the scaffold derived anti–IGF-1 antibody described in this work (Fig. 6).

## Discussion

Prior to the quantitation of IGF-1 in a native human serum, IGF-1 has to be released from its corresponding binding protein IGFBP-3. Then unwanted rebinding of IGF-1 may be prevented by adding IGF-2 in large molar excess (45). IGF-1–specific antibodies that do not cross-react with IGF-2 in serum (4, 5) and in diverse blocking and pretreatment reagent formulations are therefore a mandatory prerequisite for safe and meaningful IGF-1 diagnostics (6, 7).

Aside from target specificity, robust and reliable immunoassays certainly benefit from kinetically tailored antibodies. Ideally, antibodies used in an immunoassay exhibit binding constants with high association rates close to the diffusion limit and dissociation rates, which are rather low. Kinetic screening facilitates the selection of antibodies with suitable rate compositions (44). Interestingly, fast complex formation with mature full-length IGF-1 and IGF-2 could be achieved, even though the antibodies under study have been generated by immunization with IGF surrogates. In particular, the *K_D_* 6 pm IGF-1 binding antibody rapidly interacts with its target and constitutes a highly stable complex with a 680-min half-life. Detailed real-time kinetics of the scaffold-derived antibodies show no detectable cross-reactivity signal response at 90 nm IGF analyte concentration, and 1.3 μm IGF-2 excess does not show significant interference in the IGF-1 immunoassay.

To enable reliable immunological discrimination between IGF-1 and IGF-2, we identified poorly immunogenic but distinct IGF loop motifs and recombinantly grafted them onto SlyD FKBP scaffold protein backbones derived from *T. thermophilus* and *T. gammatolerans*. Already in the immunization campaign, IGF loop insertion-specific mice titers were obtained when using the epitope mimics instead of IGF-1 and IGF-2 as immunogens. The scaffold approach seems to be very useful for overcoming issues with poor target immunogenicity. The application of complementary proteins for immunization (*Tt*SlyD) and screening (*Tg*SlyD) campaigns even made the use of the genuine IGF-2 immunogen obsolete. In case of difficult-to-handle immunogens, sticky, poorly soluble, unstable, or host toxic proteins, the SlyD approach might be the “sly” way to successfully generate otherwise inaccessible antibodies *versus* preselected epitopes.

Commonly, the insertion of a guest domain into a host protein affects the stability of both proteins (27–29). The host protein is usually destabilized because of changes in local contacts in the region of insertion, and steric tensions may arise when the residues of the guest and the host chain ends do not match perfectly (30, 31). When comparing the near UV CD signatures of the empty scaffolds *Tg*SlyD-ΔIF and *Tt*SlyD-ΔIF with their parent proteins, the FKBP domains show structured, native-like conformations, and they do so even in the presence of the heterologous amino acid loop grafts. The chimeric fusion proteins largely maintain their structural integrity, which is a prerequisite for a sterically unhindered epitope display. The *Tt*SlyD chimeras are sufficiently stable under physiological conditions at least to a *T*_m_ of 62 °C, and the *Tg*SlyD chimeras are extraordinarily stable with melting points beyond a *T*_m_ of 80 °C. Notably, the *T. thermophilus* and *T. gammatolerans* FKBP domains are autonomously folding entities. The promoted loop accessibility is reflected by SPR real-time kinetics showing that the antibody association rate constants toward the loop grafted mimics resemble those toward the native IGFs.

When the IF domain is replaced by a target insert, the FKBP domain plays the role of a clamp, which holds the cargo in a restrained conformation. The loops are integrated into the FKBP domains via one glycine as a connector, as recommended by MD simulations. IGF-1 Gly^90^ and *Tt*SlyD Ala^122^ and Gly^123^ generate a flexible GGAG linker at the C-terminal flank of the insertion site, whereas the N-terminal site of insertion seems less flexible.

Knappe *et al*. (16) and Geitner and Schmid (21) show that a variety of structures can be accommodated by the FKBP domain in a functionally folded form (16, 21). FKBP domains from thermophiles can successfully harbor intrinsically disordered sequences, secondary structure motifs (46), and autonomously folding tertiary structures, like the 73–amino acid residue CSF1R tyrosine kinase domain (47) (data not shown here). The restriction of the method seems to be the relative orientation and distance of the graft termini rather than the number of amino acid residues to be inserted.

Because the scaffolds are cysteine-free, small proteins that can be expressed as soluble monomeric proteins in large amounts in prokaryotic expression systems, all necessary experiments from the immunization campaign to screening and quality analyses can be conducted and supplied easily. Apart from the novel immunization strategy, FKBP domains from thermophilic organisms also hold promise as powerful tools for the generation of constrained binding peptides by diverse molecular display technologies (data not shown).

Purification tags such as a C-terminally fused histidine tag can be used, because this tag does not conspicuously interfere with the folding and stability of the thermostable FKBP domains and because the insertion site is remote from the C terminus. Even though cytosolic expression already yielded soluble protein, the exceptional refolding capabilities of the chimeras pointed toward a robust matrix-assisted refolding as the method of choice to gain the FKBP-embedded immunogens in maximum yields. After purification, the scaffold variants turn out to be stable monomeric proteins. In brief, thermostable FKBP domains may serve a role as a molecular clamp into which the immunogen peptide can be fixed in a well-defined conformation but with sufficient flexibility and accessibility for presentation to the immune system. It is highly probable that immunogens of this type retain their overall structure even under the harsh conditions of standard immunization methods. This may be an advantageous feature given that in the unfavorable environment of most adjuvants, marginally stable protein immunogens tend to aggregate and thus to lose their native-like fold.

SlyD-based presentation of preselected protein domains or shorter protein fragments may be favorable in presenting putative epitopes to the immune system. Thus, defined epitopes can be grafted onto a rather generic scaffold, which otherwise would not be targeted by the immune system response, either because they are not genuinely immunodominant or because they are partially buried in the native conformation. In conclusion, thermostable SlyD FKBP domains are highly suitable as a generic scaffold platform for epitope grafting.

## Experimental procedures

### Sequences

Sequences for SlyD from *T. thermophilus* and *T. gammatolerans* were retrieved from the SwissProt database (entry Q72H58) and Uniprot database (entry C5A384), respectively. Synthetic derivatives of *Tt*SlyD and *Tg*SlyD genes were purchased from Geneart (Life Technologies) and were cloned via EcoRI and HindIII (NEB) into a T7-promotor controlled pET24a expression plasmid (Novagen). *Tt*SlyD-ΔIF, *Tt*SlyD-IGF-1(74–90), and *Tt*SlyD-IGF-2(53–65) were N-terminally tagged with a MRGS motif originating from the pQE expression vector series (Qiagen). All sequences were designed with a C-terminal GGGGSRK linker motif and histidine tags of different length for purification purposes. Sequences are listed in Table 1.

### Scaffold design and MD simulations

The X-ray crystal structure data of SlyD from *T. thermophilus* (23) (PDB accession code 3CGN) and the ^1^H-NMR-structure bundle data of human IGF-1 (24, 26) (PDB accession code 1BQT) were used. No *Tg*SlyD structural data were available at the time of this study. Modeling and MDs calculations were performed using YASARA structure software (version 13.9.8) (32, 33).

To investigate the presentation of flexible loops on the structurally stable FKBP domain of *Tt*SlyD, the flexible chaperone domain (*i.e.* the IF domain, residues 67–121, Cα distance 11.8 Å) was manually replaced by the extended IGF-1 loop structure between the short α-helices 2 and 3 (residues 25–41, Cα distance 12.0 Å). Two connection modes between the *Tt*SlyD FKBP domain scaffold and the inserted IGF-1 loop were simulated. The first linker variant consisted of a single glycine residue following His^66^ and preceding Ala^122^. The second linker comprised three amino acids, AGS following Gly^64^ and GSS preceding Gly^123^. For the simulations, starting structures were protonated using the implemented p*K_a_* prediction and hydrogen bond network optimization algorithm (34) and solvated in a periodic box (35) with its boundaries 15 Å away from the protein structure in all dimensions using constrained transferable intermolecular potential with three points (36) water molecules. The box was neutralized at pH 7.4 using 0.9% sodium chloride solution, and the water density was equilibrated to a final water density of 0.997 g/ml at 298 K. All simulations were performed utilizing the AMBER03 (37) force field for the protein residues, and the default value for electrostatic cutoff (7.86 Å) was used with Particle Mesh Ewald algorithm (35) for long-range electrostatics using 128 grid points on a 0.7 Å grid.

The structure was initially minimized (34) using first steepest descent without electrostatics to remove steric clashes, and subsequently relaxed by steepest descent minimization and simulated annealing from 298 K (time step, 2 fs; atom velocities scaled down by 0.9 every 10th step) until convergence was reached, *i.e.* the energy improved by less than 0.05 kJ/mol per atom during 200 steps. MDs calculations in an isothermal-isobaric ensemble using constrained bond length to all hydrogen atoms (36, 38) were performed at 298 K and a solvent density of 0.997 g/ml. Temperature rescaling of the atom velocities was performed using a modified Berendsen thermostat to slowly heat up the minimized system during an equilibration phase until the target temperature and density were reached. The simulation time step was 1.33 fs for intermolecular and 4 fs for intramolecular interactions; to speed up the simulation, snapshots were saved every 25 ps. The MD simulations were performed in three independent runs with different initial velocities for each system over 40 ns, and the trajectories were analyzed using YASARA (32) structure and VMD (39).

### SlyD scaffold preparation: expression and purification

*E. coli* BL21 (DE3) cells harboring the respective plasmids were grown at 37 °C in lysogeny broth medium. Expression was induced by 1 mm isopropyl-β-d-thiogalactoside for 3 h. The cells were harvested by centrifugation at 5000 × *g* for 20 min. For cell lysis, the pellet was resuspended in chilled 50 mm sodium phosphate buffer, pH 8.0, supplemented with 7 m GdmCl and 10 mm imidazole. Thereafter, the suspension was stirred on ice for 2 h. After centrifugation at 25,000 × *g* for 1 h and filtration steps using cellulose nitrate membranes at 8.0, 1.2, and finally 0.2 μm, the lysate was applied onto a nickel–nitrilotriacetic acid column equilibrated with lysis buffer. Twenty volumes of lysis buffer were applied in the washing step. To allow refolding, the GdmCl solution was slowly replaced overnight by 20 column volumes of 50 mm sodium phosphate buffer, pH 8.0, 100 mm NaCl, 10 mm imidazole, and protease inhibitor (Complete® EDTA-free, Roche Diagnostics, Germany). The protease inhibitor was removed by washing with 10 column volumes of 50 mm sodium phosphate buffer, pH 8.0, 100 mm NaCl, 10 mm imidazole. The protein was then eluted by applying 250 mm imidazole in the same buffer. Protein-containing fractions were assessed for purity by Tricine–SDS-PAGE (40).

Protein containing fractions were pooled, concentrated, and subjected to a size-exclusion chromatography (HiLoad 26/60 Superdex 200 size-exclusion chromatography column; GE Healthcare Life Sciences) in storage buffer (50 mm KH_2_PO_4_, pH 7.0, 100 mm KCl, 0.5 mm EDTA) to separate monomers from small amounts of dimers and trimers. The monomer containing fractions were recovered and assessed for purity using Novex NuPAGE SDS-PAGE gel systems (Life Technologies) under denaturing conditions and were also used for Western blotting analysis. Coomassie-like protein staining was performed using SimplyBlue Safe-Stain (Life Technologies) molecular weight marker Novex sharp standard (Life Technologies catalog no. LC5800). Protein concentration measurements were performed with a DU7400 spectrophotometer (Beckman Coulter). The molar extinction coefficients (ϵ_280_) for all proteins under study were calculated according to Pace *et al.* (41, 42).

### CD spectroscopic measurements

To investigate the structural properties of several scaffold variants, we compared the near-UV signatures obtained by CD spectroscopy of *Tt*SlyD-wt and *Tt*SlyD-ΔIF with *Tt*SlyD-IGF-1(74–90) and *Tt*SlyD-IGF-2(53–65) and of *Tg*SlyD-wt and *Tg*SlyD-ΔIF with *Tg*SlyD-IGF-2(53–65), respectively. CD spectra were recorded and evaluated using a JASCO J-720 instrument and JASCO software according to the manufacturer's recommendations. A quartz cuvette with a 0.2-cm path length was used for the measurements. For the thermal transitions, the instrument parameters were set to 1 °C resolution, 1-nm bandwidth, and a sensitivity of 20 mdeg. The sample buffer was 50 mm potassium phosphate, pH 7.0, 100 mm NaCl, 0.5 mm EDTA. Protein concentrations were as follows: 79 μm *Tt*SlyD-wt, 57 μm *Tt*SlyD-ΔIF, 65 μm *Tt*SlyD-IGF-1(74–90), 59 μm *Tt*SlyD-IGF-2(53–65), 66 μm *Tg*SlyD-wt, 52 μm *Tg*SlyD-ΔIF, and 51 μm *Tg*SlyD-IGF-2(53–65). Near-UV CD spectra were recorded at 20 °C between 250 and 330 nm with a 0.5-nm resolution and a scan speed of 20 nm/min. The spectra were accumulated nine times to improve the signal-to-noise ratio. In a subsequent experiment the CD signals were recorded as a function of temperature at a fixed wavelength of 277 nm. Melting (20 to 90 °C) and refolding (90 to 20 °C) was recorded at 1 °C/min.

### Generation of monoclonal antibodies

For the generation of IGF-1 specific antibodies, Naval Medical Research Institute mice aged 8–12 weeks received four immunizations with 100 μg of *Tt*SlyD-IGF-1(74–90) each. Similarly, for generation of IGF-2–specific antibodies, the mice were immunized with 100 μg of *Tg*SlyD-IGF-2(53–65). The immunogen was administered in a 1:2 (v/v) dilution with the respective adjuvant in a total volume of 200 μl. The first dose was injected intraperitoneally in complete Freund's adjuvant. The second and third immunizations were conducted 6 and 10 weeks after the initial immunization and administered intraperitoneally and subcutaneously, respectively, in incomplete Freund's adjuvant. Three days before spleen preparation, the final booster immunization was performed by intravenous injection of 100 μg of the respective immunogen. The mice serum titers were tested 12 weeks after the initial immunization. Serum titers of *Tt*SlyD-IGF-1(74–90) immunized mice were tested using the screening proteins *Tt*SlyD-IGF-1(74–90) and human IGF-1 (PeproTech). Titers of *Tg*SlyD-IGF-2(53–65) immunized mice were tested by a differential approach using the screening proteins *Tg*SlyD-IGF-2(53–65), *Tg*SlyD-ΔIF, *Tt*SlyD-IGF-2(53–65), and *Tt*SlyD-wt. Titers were assessed using an indirect ELISA in 96-well microtiter plates (Thermo Fisher). The wells were coated with 250 ng/ml screening protein at 4 °C overnight and then washed three times for 4 min each (Washer ELx405 Select, BioTek Instruments) with a PBS washing buffer solution comprising 0.9% (w/v) NaCl and 0.05% (w/v) Tween. The wells were blocked with 1% BSA buffer in PBS (w/v) for 1 h and then washed as previously. All further incubation steps were performed on a plate shaker at room temperature for 1 h and a filling volume of 100 μl/well. The plates were incubated with mouse serum, which was diluted 1:300 (v/v) in PBS and titrated 1:3 in eight steps. The wells were washed as previously. Bound antibodies were detected by a peroxidase-conjugated secondary antibody (peroxidase-conjugated AffiniPure F(ab′)2 fragment goat anti-mouse IgG; Dianova). ABTS (Roche Diagnostics) was finally used as a substrate, and absorption was measured with an ELISA-Reader PowerWave XS (BioTek Instruments) at 405 nm (reference wavelength, 492 nm). The measured absorption was normalized, and the half-maximum saturation was determined as a dilution factor.

Antibody-secreting, immortalized hybridoma cells were produced by the fusion of myeloma cells with murine B-cells, according to the method of Köhler and Milstein (43). Primary hybridoma cells were seeded in 96-well microtiter plates by limited dilution (44) and cultured for 1 week at 37 °C, 5% CO_2_, and 95% air humidity. Binding to the target proteins was tested by an indirect ELISA as described above. Wells with significant binding to the IGF-1 chimeric scaffold protein and below instrument threshold signal to *Tt*SlyD-ΔIF counted as positive wells and were deposited by FACS-assisted single cell sorting in 96–microtiter well plates. An indirect ELISA was performed to screen the hybridoma clones for binding activity *versus* the above mentioned target proteins. Additionally, all hybridoma clones were tested against recombinant IGF-1 (PeptroTech; 100-11) and recombinant IGF-2 (PeproTech; 100-12). Clone cultures with the respective IGF specificity were chosen for kinetic analyses. The sequence of finally selected antibodies was determined by rapid amplification of cDNA ends PCR as described (48).

### Kinetic characterization: surface plasmon resonance analyses

Kinetic screening of hybridoma primary cultures was performed according to Schräml and Biehl (44) on a Biacore 4000 instrument (GE Healthcare) at 37 °C. The system buffer was HBS-EP (10 mm HEPES, pH 7.4, 150 mm NaCl, 1 mm EDTA, 0.05% (w/v) Tween 20). Polyclonal rabbit IgG antibody RAM IgG (GE Healthcare) were immobilized via NHS chemistry at 30 μg/ml in 10 mm sodium acetate buffer, pH 4.5, at 12000 RU on a CM5 sensor. The sensor was saturated with 1 m ethanolamine. Hybridoma supernatants were diluted 1:2 in system buffer and were captured at 30 μl/min for 1 min. 150 nm of seven analyte single concentrations were injected at 30 μl/min for 2 min: 7.6-kDa human recombinant IGF-1 (PeproTech), 7.2-kDa human recombinant IGF-2 (PeproTech), 14.2-kDa *Tt*SlyD-IGF-1(74–90), 12.9-kDa *Tt*SlyD-ΔIF, 14.7-kDa *Tg*SlyD-IGF-2(53–65), 13.7-kDa *Tg*SlyD-ΔIF, and 18.6-kDa *Tg*SlyD-wt. Complex dissociation was monitored for 5 min. The sensor was regenerated by 10 mm glycine HCl solution, pH 1.7, for 2 min.

Hybridoma cultures with preferable kinetic rate composition were further cloned and analyzed at 25 °C on a Biacore T200 instrument as described above using a series S sensor chip CM5 (GE Healthcare). Clone culture supernatants were captured on the sensor surface at 10 μl/min for 2 min. The analytes were injected at 100 μl/min for 3 min in concentration series of 90, 30, 10, 3.3, 1.1, and 0 nm. The measurements were done in triplicate. Analyte dissociation was monitored for 5, 30, and 120 min, respectively. Kinetic data were evaluated using Biacore evaluation software 4.1. Antibody antigen complex half-life was calculated as described (44).

### Peptide array epitope mapping

Peptide-based epitope mappings were carried out as described and commercially offered by Intavis, Cologne, Germany using the CelluSpots^TM^ technology. Peptides 15 amino acids in length with an off-set by 1 amino acids, corresponding to the sequence of human IGF-1 or human IGF-2, were synthesized and spotted onto Intavis CelluSpots^TM^ glass slides using an Intavis slide spotting robot.

The slides were washed with ethanol and Tris-buffered saline (TBS; 50 mm Tris, 137 mm NaCl, 2.7 mm KCl, pH 8) before blocking with 5 ml of 10× Western blocking reagent (Roche Applied Science), 2.5 g of sucrose in TBS with 0.1% Tween 20 (TBST) at 4 °C for 16 h. The slides were washed with TBST and then incubated with 1 μg/ml of the corresponding IGF antibodies in TBST at ambient temperature for 2 h and washed with TBST. For detection, the slides were incubated with an anti-rabbit/anti-mouse secondary horseradish peroxidase-antibody (1:20,000 in TBST) followed by incubation with chemiluminescence substrate luminol and visualized with a LumiImager (Roche Applied Science). ELISA-positive SPOTs were quantified, and antibody-binding epitopes were identified via assignment of the corresponding peptide sequences.

### Preparation of immunoconjugates

Murine monoclonal anti–IGF-1 antibodies, derived from the scaffold and other conventional IGF-1 immunization campaigns, were produced in hybridoma cultures and purified from the cell culture supernatant. Antibodies and fragments thereof were biotinylated and ruthenylated. The lysine ϵ-amino groups of the antibodies were modified at protein concentrations of ∼10 mg/ml with *N*-hydroxy-succinimide activated biotin and ruthenium labels, respectively. The label/protein molar ratio varied from 2:1 to 5:1, depending on the respective antibody fragments. The reaction buffer was 150 mm sodium phosphate (pH 8.0), 50 mm NaCl, 1 mm EDTA. The reaction was carried out at room temperature for 15 min and was stopped by adding buffered l-lysine to a final concentration of 10 mm. To avoid hydrolytic inactivation of the labels, the respective stock solutions were prepared in dried DMSO (SeccoSolv®, Merck). DMSO concentrations up to 15% in the reaction buffer were well-tolerated by all antibody variants studied. After the coupling reaction, unreacted free label was removed by passing the crude protein conjugate over a gel-filtration column (Superdex 200 HiLoad).

### ECLIA measurements

For the quantification of recombinant human IGF-1 in presence of increasing IGF-2 concentrations, an Elecsys® cobas e 411 immunoanalyzer (Roche Diagnostics) was used. The immunoassay utilizes the sandwich principle. An immunocomplex with IGF-1 is formed by two anti–IGF-1 mAb conjugates; the IGF-1 loop binding antibody was obtained in the scaffold immunization campaign described in this work. The second monoclonal anti–IGF-1 antibody binds IGF-1 at a different epitope and is beyond the scope of this work.

Signal detection in the Elecsys® immunoanalyzers is based on electrochemiluminescence. Biotinylated capture antibodies are immobilized on the surface of streptavidin-coated magnetic beads, which are magnetically focused on a platinum electrode. In the presence of IGF-1, a ternary immune complex consisting of the biotinylated antibody, the IGF-1 analyte, and the ruthenylated antibody is formed. Upon electric excitation and in the presence of tripropylamine, switching of the ruthenium cation between the redox states 2+ and 3+ leads to light emission at 620 nm. The signal output is in arbitrary light units (counts). A native human serum sample was spiked with human recombinant IGF-1 (Roche, Penzberg) to a target concentration of 1 μg/ml and was split into two aliquots, A and B. Aliquot A was additionally spiked with 4 μg/ml IGF-2. Aliquot B was spiked with Elecsys® diluent universal accordingly to keep equivalent volumes in aliquot A and B. Aliquot A and B were then diluted with each other to ensure a constant IGF-1 concentration of 1 μg/ml and an IGF-2 concentration dilution series of 8000, 6000, 5000, 4500, 4250, and 4000 ng/ml. The samples were measured in triplicate.

## Author contributions

C. P., C. S., D. C., F. K., M. B., and M. S. conceptualization; C. P., C. S., M. B., and M. S. software; C. P., C. S., D. C., M. G., and M. S. supervision; C. P., C. S., D. C., M. G., F. K., M. B., L. v. P., S. G., H. K.-S., A. H., and M. S. validation; C. P., C. S., D. C., H. D., M. G., F. K., M. B., L. v. P., S. G., H. K.-S., A. H., and M. S. investigation; C. P., C. S., D. C., H. D., F. K., M. B., L. v. P., S. G., H. K.-S., A. H., and M. S. methodology; C. P., C. S., and M. S. writing-original draft; C. P., C. S., H. D., M. G., F. K., M. B., L. v. P., S. G., H. K.-S., and M. S. writing-review and editing; M. G. and M. S. resources; M. G., A. H., and M. S. project administration; M. B. and M. S. data curation; M. S. formal analysis; M. S. funding acquisition; M. S. visualization.

## Supplementary Material

Supporting Information

## Footnotes

2 The abbreviations used are:

IGF: insulin-like growth factor
FKBP: FK-506–binding protein
IF: insert-in-flap
MD: molecular dynamic
ECLIA: electrochemiluminescence immunoassay
PDB: Protein Data Bank
TBS: Tris-buffered saline
SlyD: sensitivity to lysis D.
