# Supplementary material for: A novel epitope-presenting thermostable scaffold for the development of highly specific insulin-like growth factor-1/2 antibodies
Source: J Biol Chem. 2019 Jul 23;294(36):13434–44. doi: 10.1074/jbc.RA119.007654 (PMC6737233; doi:10.1074/jbc.RA119.007654)
Supplement: Supporting Information [file supp_294_36_13434__index.html]

A novel epitope-presenting thermostable scaffold for the development of highly specific IGF-1/-2 antibodies — Thermostable SlyD: a scaffold module in antibody development — A novel epitope-presenting thermostable scaffold for the development of highly specific insulin-like growth factor-1/2 antibodies — Thermostable SlyD: a scaffold module in antibody development — Supporting Information 

# A novel epitope-presenting thermostable scaffold for the development of highly specific insulin-like growth factor-1/2 antibodies

## Supporting Information

- Supporting Information (to be published online) - Supporting Information (to be published online)
