# Supplementary material for: A novel epitope-presenting thermostable scaffold for the development of highly specific insulin-like growth factor-1/2 antibodies
Source: J Biol Chem. 2019 Jul 23;294(36):13434–44. doi: 10.1074/jbc.RA119.007654 (PMC6737233; doi:10.1074/jbc.RA119.007654)
Supplement: Supporting Information [file supp_RA119.007654_143255_1_supp_340851_psvn0p.docx]

**Supporting Information**

**Supplementary Table 1
Production yields after refolding and purification of the *Tt*SlyD and *Tg*SlyD wild type proteins and their derivatives**

| **Scaffolds** | **Biomass (wet pellet), g** | **Protein yield after on-column refolding and Ni-NTA, mg** | **Protein yield after SEC, mg** | **Final formulation concentration, mg/mL** |
| --- | --- | --- | --- | --- |
| *Tt*SlyD-wt | 6.3 | 129 | 23 | 5.2 |
| *Tt*SlyD-ΔIF | 7.2 | 208 | 54 | 5.4 |
| *Tt*SlyD-IGF-1(74–90) | 6.2 | 169 | 40 | 5.0 |
| *Tt*SlyD-IGF-2(53–65) | 7.8 | 24 | 13 | 5.2 |
| *Tg*SlyD-wt | 5.7 | 80 | 23 | 2.3 |
| *Tg*SlyD-ΔIF | 5.0 | 67 | 17 | 1.7 |
| *Tg*SlyD-IGF-2(53–65) | 6.3 | 144 | 27 | 2.7 |

IF, insert-in-flap; IGF, insulin-like growth factor; NTA, nitrilotriacetic acid; SEC, size exclusion chromatography; SlyD, sensitivity to lysis D; *Tg*SlyD-wt, *Thermococcus gammatolerans* SlyD wild type; *Tt*SlyD-wt, *Thermus thermophilus* SlyD wild type

**Supplementary Table 2
Antibody titers (relative units) of NMRI mouse sera after immunization**

| ***Tt*SlyD-IGF-1(74–90)** | | | ***Tg*SlyD-IGF-2(53–65)** | | | | |
| --- | --- | --- | --- | --- | --- | --- | --- |
| **Mouse** | **IGF-1** | ***Tt*SlyD-IGF-1(74–90)** | **Mouse** | ***Tg*SlyD-IGF-2(53–65)** | ***Tg*SlyD-ΔIF** | ***Tt*SlyD-IGF-2(53–65)** | ***Tt*SlyD-wt** |
| 1 | 2500 | 26000 | 1 | 67000 | 69000 | 1700 | 600 |
| 2 | 4700 | 50000 | 2 | 51000 | 54000 | 1800 | 700 |

IF, insert-in-flap; IGF, insulin-like growth factors; NMRI, Naval Medical Research Institute; SlyD, sensitivity to lysis D; *Tg*SlyD, *Thermococcus gammatolerans* SlyD; *Tt*SlyD, *Thermus thermophilus* SlyD; wt, wild type


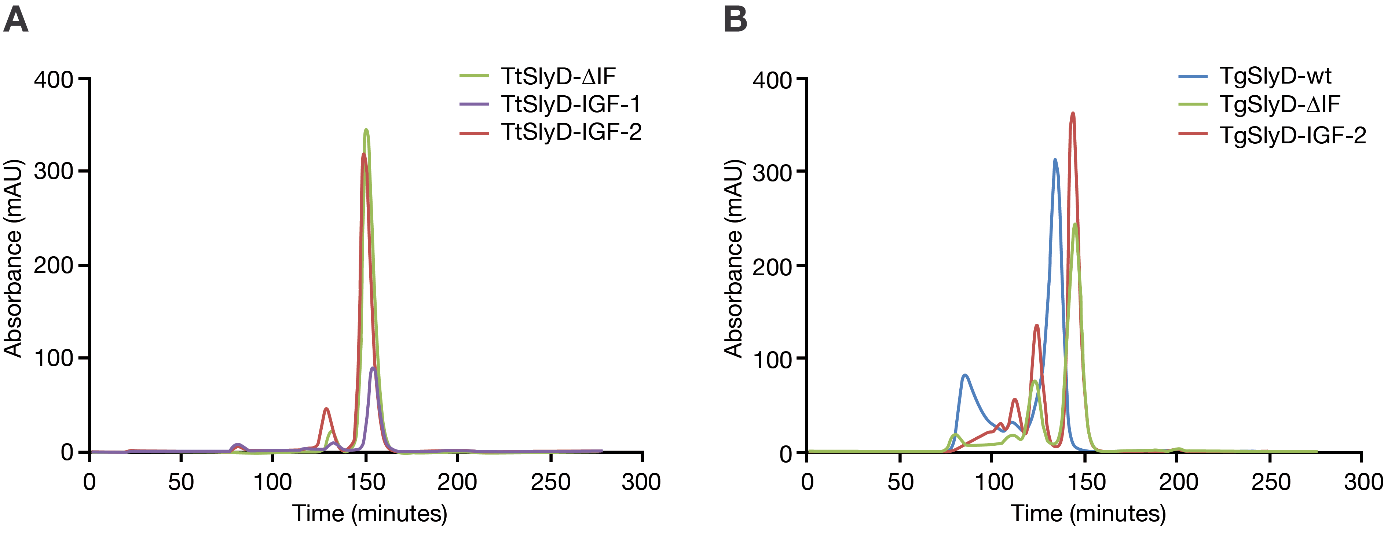


**Supplementary Figure 1. Purification of *Tt*SlyD and *Tg*SlyD scaffold derivatives by SEC.** The example SEC profiles illustrate the purification of *Tt*SlyD and *Tg*SlyD scaffold proteins. The dominating peak at 150 min, containing the monomeric fusion protein, was fractionated. (A) SEC profiles of protein *Tt*SlyD-ΔIF and fusion proteins *Tt*SlyD-IGF-1(74–90) and *Tt*SlyD-IGF-2(53–65). (B) SEC profiles of protein *Tg*SlyD-wt, *Tg*SlyD-ΔIF and fusion protein *Tg*SlyD-IGF-2(53–65).
IF, insert-in-flap; IGF, insulin-like growth factor; SlyD, sensitivity to lysis D; *Tg*SlyD, *Thermococcus gammatolerans* SlyD; *Tt*SlyD, *Thermus thermophilus* SlyD; wt, wild type


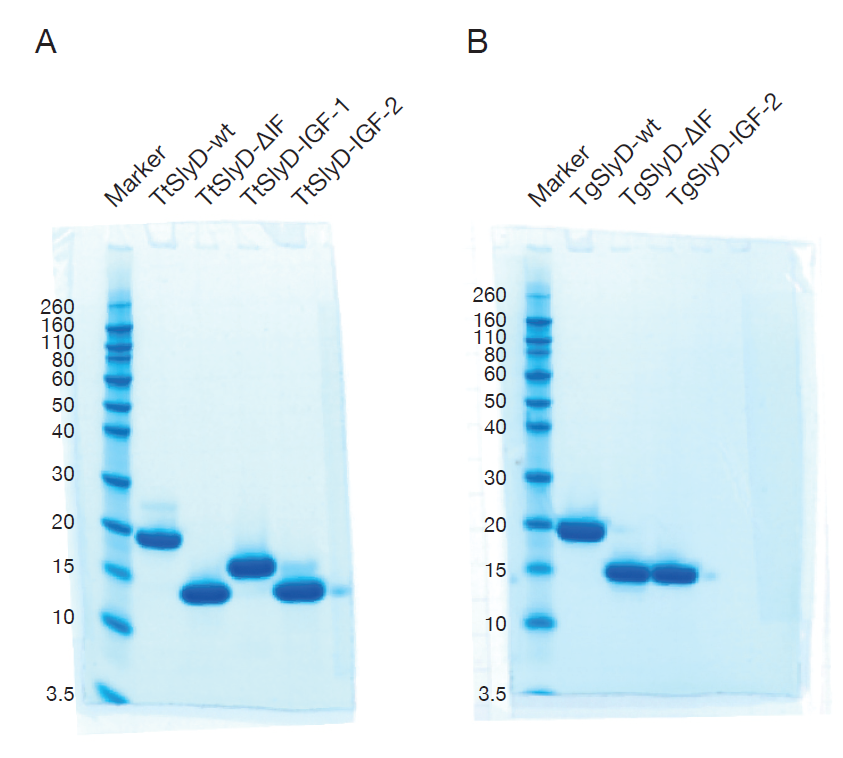


**Supplementary Figure 2. 10% SDS PAGE coomassie stained gels of SlyD wildtype and derivative proteins after Ni-NTA and SEC purification.** (A) *Tt*SlyD-wt, *Tt*SlyD-ΔIF, *Tt*SlyD-IGF-1 and *Tt*SlyD-IGF-2. (B) *Tg*SlyD-wt, *Tg*SlyD-ΔIF and *Tg*SlyD-IGF-2.
IF, insert-in-flap; IGF, insulin-like growth factor; SlyD, sensitivity to lysis D; *Tg*SlyD, *Thermococcus gammatolerans* SlyD; *Tt*SlyD, *Thermus thermophilus* SlyD; wt, wild type


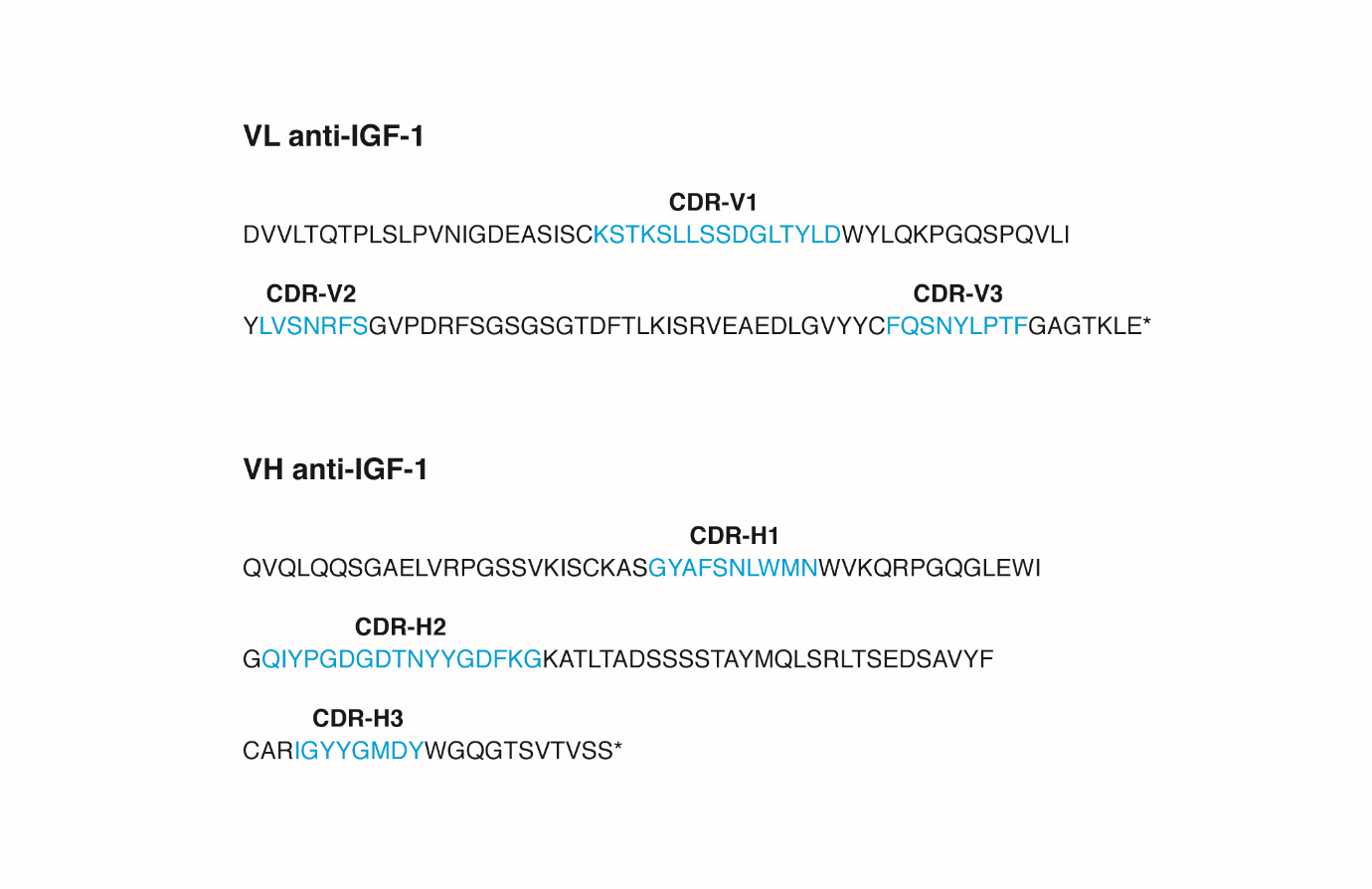


**Supplementary Figure 3. Anti IGF-1 antibody VL and VH chain sequences.** Complementarity-determining regions indicated by blue text.
CDR, complementarity-determining region; IGF, insulin-like growth factor; VH, heavy chain variable domain; VL, light chain variable domain.


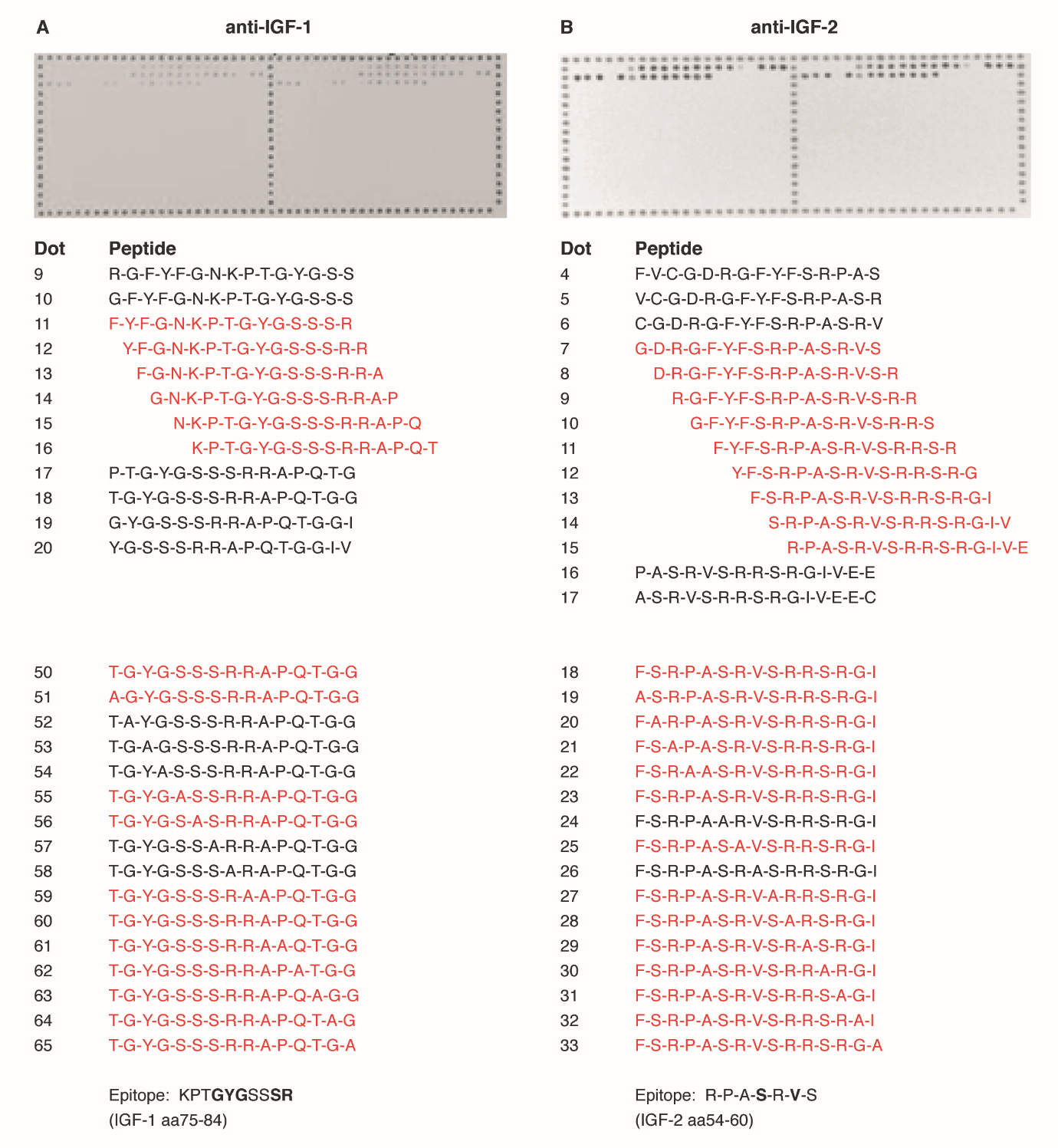


**Supplementary Figure 4. Peptide-based 2D epitope mapping of anti-IGF-1 and anti-IGF-2 antibodies.**
